# Anti-amyloid therapies work for Alzheimer’s disease

**DOI:** 10.1093/braincomms/fcad204

**Published:** 2023-07-17

**Authors:** John Hardy

**Affiliations:** Reta Lilla Weston Research Laboratories and Department of Neurodegenerative Disease and Dementia Research Institute, UCL Institute of Neurology, London WC1N 3BG, UK

Anti-amyloid therapies slow Alzheimer disease progression: with the Federal Drug Administration approval of lecanemab and with the reported press release of the data from the donanemab trial, the argument about whether these agents slow disease is now settled. The framework of understanding proposed by Karran and De Strooper^1^ seems to fit all the published data.^2^ This leads to a predictive model for other anti-amyloid drugs and that too is extremely valuable going forward. The review by Liu *et al.*^3^ in *Brain Communications* makes clear points about the limitations of the current agents, and these concerns have led to appropriate restrictions on use: few would currently disagree with these restrictions, and it will be interesting to see if the open extension label data on the lecanemab trial and the release of the primary data from the donanemab trial alleviate some of these concerns. These restrictions and concerns should not be conflated, however, with the primary outcome of the trials: the drugs work and that is a cause for celebration!

When I wrote the amyloid hypothesis in 1991^4^ contemporaneously with similar articles from Glenner,^5^ from Bush *et al.*,^6^ and from Selkoe.^7^ I wrote my views based on the data available at that time. These data were from the molecular pathology analyses of others and from our own genetic analyses. Castellani and Perry,^8^ long campaigners against the amyloid hypothesis, criticize our ideas as Teflon hypotheses implying we have been wrong to change our ideas (though I suggest rereading Hardy and Allsop^4^ because it remains close to my current views). I make no apology whatsoever for (reasonably subtly) changing my mind over the intervening 30 years. With my colleagues, I have been trying to gain a deeper understanding of the disease pathogenesis: identifying tau mutations in tangle diseases,^9^ crossing amyloid mice with tau mice to show that amyloid is upstream of tangles,^10^ and identifying *triggering receptor expressed on myeloid cells 2* mutations^11^ drawing microglia into the circle of pathogenesis. Of course, my ideas have changed: if they had not, I would have been wasting my time!

There is a lot still to do: on early and accurate detection of disease, on developing easier to use anti-amyloid regimens and on identifying and prosecuting new targets: with all this work to do, we should not waste our time arguing about whether amyloid has been a legitimate or successful disease target. Clearly, it was.

## Data Availability

Data sharing is not applicable to this article as no new data were created or analysed.
